# Biomarkers as diagnostic or prognostic indicators of delirium: examining the current evidence

**DOI:** 10.12968/bjnn.2025.0007

**Published:** 2025-10-02

**Authors:** E Emery, O Hamer, J Hill

**Affiliations:** Lancashire Teaching Hospitals NHS Foundation Trust; University of Central Lancashire

**Keywords:** Delirium, Diagnosis, Biomarkers, Systematic review, Pathophysiology, Dementia, Neurocognitive

## Abstract

Delirium is a substantial global health concern. Delirium can lead to longer hospital stays and increased healthcare costs. Effective detection and prevention of delirium is still a major challenge for health-care organisations globally. This is largely because the cause(s) of the condition are still unknown. There are multiple factors which may contribute to the aetiology of delirium and a range of neurobiological processes that may be associated with its pathophysiology. With this said, evidencing these processes is a significant challenge as there is a dearth of existing methods of identification. Recently, the use of biomarkers has become a popular method in the identification of delirium and its risk of development. The identification of biomarkers associated with delirium may provide insight into its pathophysiology and aid in diagnosis and management. However, there is a lack of research that has synthesised the diagnostic and prognostic value of biomarkers associated with delirium, and how they can be employed to improve patient outcomes. A systematic review by Dunne et al. 2021 was undertaken to explore this association of biomarkers and delirium. This commentary aims to critically appraise the methods used within the review by Dunne et al. (2021) and expand upon the findings in the context of clinical practice.

## Introduction

Delirium is a substantial global health concern that affects more than 16 percent of hospital inpatients (Reynish et al. 2017; Shenkin et al. 2019). Delirium has been defined as an acute and fluctuating neuropsychiatric condition (Inouye et al. 1990; Shenkin et al. 2019). The condition often occurs in people who are medically unwell, due to underlying conditions which has put them at risk (e.g., dementia, cancer, infection, and renal impairment) (Wilson et al. 2020). The condition is associated with increased risk of morbidity and mortality (Hughes et al. 2021; Inouye 2006), as well as long-term cognitive decline (Goldberg et al. 2020). Delirium can also lead to longer hospital stays and increased healthcare costs (Dziegielewski et al. 2021; van Lieshout et al. 2022; Webber et al. 2021). Effective detection and prevention of delirium is still a major challenge for health-care organisations globally, largely because the cause(s) of the condition are still unknown (Wilson et al. 2020).

Within the literature, there are several proposed mechanisms as to the cause(s) of delirium (Gunther et al. 2008; Hshieh et al. 2008; Maldonado 2018). Firstly, some researchers propose that reduced brain efficiency, particularly in subcortical regions may prompt delirium (Lozano-Vicario et al. 2023; van Montfort et al. 2019). A further mechanism may be a consequence of impaired connectivity within brain networks which can disrupt neurotransmitter balance in cholinergic and noradrenergic neurons (leading to episodes of delirium) (Hshieh et al. 2008; Morandi et al. 2012). Other research proposes that delirium may be caused by neuroinflammatory responses and alterations in glial cells (from illness) leading to exaggerated pro-inflammatory reaction (Murray et al. 2012). Researchers have also proposed that there may be disruptions within the interaction between the dorsolateral prefrontal cortex and the posterior cingulate cortex, which play a role in the default mode network (Choi et al. 2012). Given that multiple factors may contribute to the aetiology of delirium, it is likely that there are a range of neurobiological processes that may be associated with its pathophysiology (Lozano-Vicario et al. 2023). With this said, evidencing these processes is a significant challenge, as there is a dearth of existing methods of identification (Dunne et al. 2021).

Recently, the use of biomarkers has become a popular method in the identification of delirium and its risk of development (Lozano-Vicario et al. 2023). Biomarkers have been described as any substance, structure, or process that can be measured within the body (or its products) that has the capacity to influence or predict the occurrence of a specific outcome or disease (Califf 2018). Some examples of potential biomarkers that may be associated with delirium include inflammatory mediators (e.g., interleukin-1, IL-6 and interferon) insulin like growth factor-1, genetic markers, and serum anticholinergic activity (detectable in blood, other bodily fluids, or tissues) (Khan et al. 2011; Maldonado 2018). The identification of biomarkers associated with delirium may provide insight into the pathophysiology and aid in its diagnosis and management (Yu et al. 2023). However, there is a lack of research that has synthesised the diagnostic and prognostic value of biomarkers associated with delirium, and how they can be employed to improve patient outcomes (Dunne et al. 2021).

Further research is needed to inform healthcare professionals of the progress in this area and provide guidance on if it holds implications for clinical practise. A systematic review by Dunne et al. 2021 was undertaken to explore this association of biomarkers and delirium. This commentary aims to provide an up-to-date synthesis of the diagnostic and prognostic value of existing biomarkers associated with delirium by critically appraising a recent systematic review (Dunne et al. 2021).

## Methods of Dunne et al, (2021)

A comprehensive search of five databases (including MEDLINE, EMBASE, CINAHL, Web of Science and the COCHRANE library) was undertaken for eligible studies published from January 2000 to June 2019. The search strategy was limited to studies published in English. The review included studies investigating a relationship between biomarkers and delirium, where delirium was assessed using a validated assessment tool. The inclusion criteria also stated that any study methodology was included if it led to publication in the topic of interest. However, studies were excluded if they included case reports, abstracts, editorials, studies involving delirium tremens, or animal studies.

The methods of screening and data extraction were largely unclear within the systematic review. There was an indication that study selection and data extraction were carried out in duplication, however this could not be confirmed. In addition, no assessment of bias or critically appraisal of included studies was conducted. However, the review was conducted in accordance with the Meta-Analysis of Observational Studies in Epidemiology guidelines.

## Results of Dunne et al, (2021)

From the multi-database search, 2082 studies were identified. Following full screening, 73 were included in the review. The 73 studies reported on a total of 14 biomarkers associated with delirium. Studies included biomarkers (singly or combinations) from cerebrospinal fluid, serum, or plasma.

The below table presents the findings of each individual biomarker that was studied in the review. This table is based upon the data given in appendix A and supplementary tables 2 to 15.

**Table:** 

Biomarkers	How many studies examined the biomarkers	Order	Total number of participants within studies	Number of studies found a statistically significant positive association with delirium	Number of studies found a statistically significant negative association with delirium	Positive association with delirium with comorbidity	Negative association with delirium with comorbidity	Missing data/non-significant association	Notes/external validity
SAA	6	8	647	0	3	0	0	3 - non-significant.	5 studies used serum samples, 1 used plasma samples. 2 studies took place in medical settings, 4 studies took place in surgical settings
All Amino Acids *	9		942	6	5	1	0	3	4 studies took place in a medical setting, and 5 studies took place in surgical settings.
HVA	3	14		1	0	0	0	2	
Tryptophan	6	9							
Tryptophan:LNAA ratios	4	12		0	4	0	0	0	1 study used CSF samples, 6 studies used serum samples, and 2 studies used both CSF and serum samples combined.
Phenylalanine: LNAA ratios	3	15		3	0	0	0	0	
Melatonin	3	16	99	1	1	1	0	0	All 3 studies used serum samples. 2 studies took place in surgical settings, 1 study took place in a medical setting.
IL-6	23	1	2,543	14	0	0	0	9 7-insignificant data 1-missing data as IL-6 levels above detection limit associated with delirium. 1-insignificant after adjusting for pre-existing cognitive impairment.	17 studies used serum samples, 2 studies used plasma samples, 1 study used blood samples, 2 studies used CSF and serum samples, 1 study used plasma and CSF samples. 15 studies took place in surgical settings, 8 studies took place in medical settings.
IL-8	7	7	669	5	0	0	0	2: 1-insignificant data after adjusting for co-morbidities. 1-missing data as IL-8 levels above the detection limit associated with delirium.	6 studies used serum samples, 1 used plasma and CSF samples. 2 studies took place in medical settings, 5 studies took place in surgical settings.
IL-1	5	11	360	2	0	0	0	2—missing data as IL-1 levels were below the detection limit. 1-insignificant data.	4 studies used serum samples, 1 study used serum and CSF samples. 2 studies took place in medical settings, 3 studies took place in surgical settings.
IL-2	4	13	371	3	0	1	0	1	All 4 studies used serum samples. All 4 studies took place in surgical settings.
CRP	17	2	3,501	7	0	4	0	6: 5-insignificant data. 1-insignificant data after adjusting for confounding variables.	4 studies used plasma samples, 12 studies used serum samples, 1 study used CSF and serum samples. 9 studies took place in surgical settings, 8 studies took place in medical settings.
IGF-1	10	5	943	1	5	0	0	4-insignificant data.	1 study used plasma samples, 8 studies used serum samples, 1 study used serum and CSF samples. 5 studies took place in surgical settings, 5 studies took place in medical settings.
S-100B	10	4	1,066	6	0	0	0	4-insignificant data.	8 studies used serum samples, 1 study used blood samples, 1 study used CSF samples. 4 studies took place in surgical settings, 6 studies took place in medical settings.
APO-E	6	10	825	0	0	1	0	5-insignificant data.	5 studies used serum samples, 1 study used whole blood samples. 4 studies took place in surgical settings, 2 studies took place in medical settings.
Cortisol	11	3	1,279	8	0	0	0	3 - insignificant data.	9 studies used serum samples, 1 study used plasma samples, 1 study used serum and CSF samples. 8 studies took place in surgical settings, 3 studies took place in medical settings.
Estradiol	1	17	141	1	0	0	0	0	This study used serum samples. This study took place in a medical setting.
TNF-alpha	9	6	812	5	0	0	0	4 2-insignificant data. 2-missing data as levels below the detection limit.	7 studies used serum samples, 1 study used blood samples, 1 study used plasma and CSF but TNF was only measured in the CSF. 5 studies took place in surgical settings, 4 studies took place in medical settings.

Abbreviations: SAA = Serum Anticholinergic Activity, HVA = Homovanillic Acid, LNAA = large neutral amino acids, IL = Interleukin, CRP = C-Reactive Protein, IGF-1 = Insulin-like Growth Factor-1, APO-e = Apolipoprotein-E, Tumour Necrosis Factor- Alpha (TNF-alpha).

## Commentary

Using the modified JBI critical appraisal checklist, the review satisfied four of nine criteria. This systematic review had several limiting factors such as the dearth of information related to the study selection process, inclusion criteria and lack of critical appraisal of included studies.

The inclusion criteria were deemed inappropriate due to one criterion that stated any study methodology could be included if it led to publication within the topic of interest. This criterion was inconsistent to the other inclusion and exclusion requirements. A further limitation was the lack of investigation of publication bias throughout the paper, suggesting that the likelihood of this bias was not assessed. Furthermore, specified sections of this systematic review were deemed unclear such as the methods used to minimise data extraction errors (i.e., two independent authors conducting the study selection process). Therefore, the findings of this systematic review should be viewed with some caution.

### Summary of evidence

The findings of this review highlight that biomarkers such as IL-6, IL-2, IL-8, TNF-alpha, estradiol, cortisol, tryptophan and S-100B may be positively associated with delirium, which could be important diagnostic or prognostic indicators. The evidence is currently unclear as to whether other biomarkers such as CRP, IGF-1, IL-1 and melatonin can be utilised for diagnostic or prognostic purposes related to delirium. The evidence also suggests that there was largely no association between biomarkers of HVA, SAA, APO-E and delirium, suggesting that these may not have a utilisation as diagnostic or prognostic indicators. That being said, many of these associations are confounded by a multitude of factors and as such, further research is needed to better understand the role of these biomarkers in delirium (Dunne et al. 2021).

### Associations between biomarkers and delirium

The findings from the review suggest that disturbances in neurotransmitter pathways as indicated by biomarkers such as tryptophan that is used in the biosynthesis of proteins (in relation to delirium), may play some role in the pathogenesis of delirium. This is consistent with previous evidence that has highlighted imbalances in levels of neurotransmitters may impact upon cognitive function, memory, attention, and sleep-wake cycles, which could precipitate delirium (Maldonado 2018). Future research assessing levels of neurotransmitters in the cerebrospinal fluid or blood may provide insights into how specific neurochemical imbalances may impact on delirium.

Although previous research has shown associations between cholinergic deficiency and delirium, the findings of this review do not support these relationships (Hshieh et al. 2008). The inconsistency in findings may be due to differences in detection methods or the absence of consideration for pre-existing confounders which have been known to impact on delirium (i.e., cognitive dysfunction and age) (Nitchingham and Caplan 2021). As only 12 out of the 23 studies adjusted for potential moderating factor of comorbidities. The findings of this review also oppose previous findings that cholinergic activity is linked to anti-inflammatory pathways (increased inflammatory response) which may contribute to the pathogenesis of delirium (Dunne et al. 2021). That said, further research is needed to better understand how moderating factors may impact on these biomarkers and their association with delirium.

Previous research has proposed that cortisol may disrupt the hypothalamic-pituitary-axis due to acute stress and this can lead to cognitive dysfunction linked to delirium (Jones and Gwenin 2021; Thau et al. 2023). The findings of this review support this research showing that that elevated cortisol levels may be associated with delirium, and that identification could assist clinicians in early detection of delirium (Dunne et al. 2021). That said, further research is still needed prior to implementation of routine cortisol assessments, as it is likely that comorbidities may impact on the relationship between cortisol and delirium (Dunne et al. 2021).

Alongside other biomarkers, the current review shows that elevated levels of estradiol may be associated with delirium, even after adjusting for other confounding factors (Dunne et al. 2021). However, this was based upon only a single study. Although this is consistent with previous literature which has shown that estradiol may impact on cognitive dysfunction, a precursor to delirium (Luine 2014). Neuroscience nurses and neurologists could consider this association when assessing, evaluating, and managing patients, particularly those pre-surgery (as a better understanding of estradiol levels may lead to early diagnosis of delirium). That said, further research is needed to explore the complexities of this relationship and to refine clinical practice guidelines prior to implementation into standard operating procedures.

### Implications for Clinical Practice

The findings of the review by may Dunne et al (2021) offer some tentative insights for neurologists and neuroscience nurses when attempting to prevent and manage delirium. Biomarkers that have shown a positive association with delirium, such as IL-6, IL-2, IL-8, TNF-alpha, estradiol, cortisol, tryptophan, and S-100B, may serve as potential diagnostic or prognostic indicators. The early detection of potential episodes of delirium through biomarker indicators, may serve to guide clinicians in implementing tailored interventions to improve patient outcomes. That said, neurologists and neuroscience nurses should be cautious when interpreting the role of biomarkers as much of the evidence is inconsistent and limited in scope (e.g., study design and methodological quality). Given the limitations with current evidence, firm recommendations for the implementation of biomarker assessment for patients at risk of delirium cannot yet be made. Further research is needed to clarify the relevance of biomarkers for diagnostic and prognostic applications for delirium. Given the promising literature relating to some specific biomarkers (e.g., inflammatory markers of IL-6, IL-2, IL-8, TNF-alpha, estradiol, cortisol, tryptophan and S-100B), neurologists and neuroscience nurses should continuously monitor literature and adapt clinical practice to new emerging evidence.

### Implications for future research

One of the key concerns of research focused in this area is the uncertainty regarding the pathophysiological mechanism of delirium, and the dearth of effective interventions to treat the condition (Oh et al. 2017; Yu et al. 2023). The current systematic review highlights conflicting evidence on the associations between delirium and various biomarkers. Previous literature has stated that inconsistencies in these findings (relating to associations) may be due to the fact that observational studies can be exposed to a range of bias as a consequence of unmeasured confounding and reverse causation (Yu et al. 2023). To tackle these concerns and better understand the associations between biomarkers and delirium, future research should utilise prospective study designs, improve methodological quality, mitigate bias, and ensure greater sample sizes (to attain generalisability of the results).

Given that a large proportion of research has relied upon the investigation of cerebrospinal fluid (CSF), further research should include additional diagnostic modalities, including human peripheral blood sampling, which may provide a more comprehensive view of delirium’s pathophysiology (through analysis of platelets, neutrophils, microparticles, monocytes and rare circulating cells) (Dagur and McCoy 2015). To support these sampling methods, future research should also employ other diagnostic techniques like electroencephalogram (brain scan known for its diagnostic benefit in delirium) to compliment blood and plasma samples, which may confirm associations between specific biomarkers and delirium (Taylor et al. 2022). Given the significant constraints outlined in this review, a forthcoming examination should make it a priority to tackle these challenges. Specifically, there is a need to ensure that a thorough critical evaluation of all incorporated studies is conducted and leveraged in the interpretation of the evidence.

## Conclusion

In summary, the findings highlight that associations between specific biomarkers and delirium may provide some benefit for diagnostic or prognostic purposes. However, although there are some promising findings within existing literature, current limitations within the evidence mean that recommendations for implementation into clinical practice cannot yet be made. The pathophysiology of delirium is complex with a range of factors, and more research is needed to better understand the interplay of these factors and how biomarkers associated with delirium may aid in the diagnosis and prognosis of the condition.

### CPD reflective questions

- Which of the biomarkers analysed in the review show promise for diagnostic or prognostic purposes of delirium?
- What processes would need to be in place for this data to be regularly available for clinicians and practice within your own organisation?
- How would you use the data of these biomarkers within your own practice?

## Figures and Tables

**Table 1:** Critical appraisal of the systematic review using the JBI Checklist for Systematic Reviews

JBI items	Responses
1. Is the review question clearly and explicitly stated?	Yes, the review described the PICOs in adequate detail.
2. Were the inclusion criteria appropriate for the review question?	No, the review stated that any study can be included in the review if it leads to publication in the topic of interest.
3. Was the search strategy appropriate?	Yes, search strategy included relevant databases and terms for the search.
4. Were the sources and resources used to search for studies adequate?	Yes, a systematic literature search was conducted from multiple bibliographic databases.
5. Were the criteria for appraising studies appropriate?	No, the review did not conduct a critical appraisal of included studies.
6. Was critical appraisal conducted by two or more reviewers independently?	No, critical appraisal of included studies was not assessed.
7. Were there methods to minimize errors in data extraction?	Unclear, independent multi author data extraction could not be confirmed.
8. Were the methods used to combine studies appropriate?	Yes, studies were pooled with meta-analysis.
9. Was the likelihood of publication bias assessed?	No, the review did not explore publication bias
Total	Score 4/9

## Key Points

- Biomarkers such as IL-6, IL-2, IL-8, TNF-alpha, estradiol, cortisol, tryptophan and S-100B may be associated with delirium.
- The evidence is currently unclear as to whether other biomarkers such as CRP, IGF-1, IL-1 and melatonin can be utilised for diagnostic or prognostic purposes related to delirium.
- The evidence suggests that it is unlikely that there are associations between biomarkers of HVA, SAA, APO-E, and delirium.
- Further research is needed to explore the complexities of associations between biomarkers and delirium before recommendations to practice can be made.

## References

[R1] Califf RM (2018). Biomarker definitions and their applications. Exp Biol Med (Maywood).

[R2] Choi SH, Lee H, Chung TS, Park KM, Jung YC, Kim SI, Kim JJ (2012). Neural network functional connectivity during and after an episode of delirium. Am J Psychiatry.

[R3] Dagur PK, McCoy JP (2015). Collection, storage, and preparation of human blood cells. Curr Protoc Cytom.

[R4] Dunne SS, Coffey JC, Konje S, Gasior S, Clancy CC, Gulati G, Meagher D, Dunne CP (2021). Biomarkers in delirium: A systematic review. Journal of Psychosomatic Research.

[R5] Dziegielewski C, Skead C, Canturk T, Webber C, Fernando SM, Thompson LH, Foster M, Ristovic V, Lawlor PG, Chaudhuri D (2021). Delirium and associated length of stay and costs in critically ill patients. Crit Care Res Pract.

[R6] Goldberg TE, Chen C, Wang Y, Jung E, Swanson A, Ing C, Garcia PS, Whittington RA, Moitra V (2020). Association of delirium with long-term cognitive decline: A meta-analysis. JAMA Neurol.

[R7] Gunther ML, Morandi A, Ely EW (2008). Pathophysiology of delirium in the intensive care unit. Crit Care Clin.

[R8] Hshieh TT, Fong TG, Marcantonio ER, Inouye SK (2008). Cholinergic deficiency hypothesis in delirium: A synthesis of current evidence. J Gerontol A Biol Sci Med Sci.

[R9] Hughes CG, Hayhurst CJ, Pandharipande PP, Shotwell MS, Feng X, Wilson JE, Brummel NE, Girard TD, Jackson JC, Ely EW (2021). Association of delirium during critical illness with mortality: Multicenter prospective cohort study. Anesth Analg.

[R10] Inouye SK (2006). Delirium in older persons. N Engl J Med.

[R11] Inouye SK, van Dyck CH, Alessi CA, Balkin S, Siegal AP, Horwitz RI (1990). Clarifying confusion: The confusion assessment method. A new method for detection of delirium. Ann Intern Med.

[R12] Jones C, Gwenin C (2021). Cortisol level dysregulation and its prevalence-is it nature’s alarm clock?. Physiol Rep.

[R13] Khan BA, Zawahiri M, Campbell NL, Boustani MA (2011). Biomarkers for delirium--a review. J Am Geriatr Soc.

[R14] Lozano-Vicario L, García-Hermoso A, Cedeno-Veloz BA, Fernández-Irigoyen J, Santamaría E, Romero-Ortuno R, Zambom-Ferraresi F, Sáez de Asteasu ML, Muñoz-Vázquez ÁJ, Izquierdo M (2023). Biomarkers of delirium risk in older adults: A systematic review and meta-analysis. Front Aging Neurosci.

[R15] Luine VN (2014). Estradiol and cognitive function: Past, present and future. Horm Behav.

[R16] Maldonado JR (2018). Delirium pathophysiology: An updated hypothesis of the etiology of acute brain failure. Int J Geriatr Psychiatry.

[R17] Morandi A, Rogers BP, Gunther ML, Merkle K, Pandharipande P, Girard TD, Jackson JC, Thompson J, Shintani AK, Geevarghese S (2012). The relationship between delirium duration, white matter integrity, and cognitive impairment in intensive care unit survivors as determined by diffusion tensor imaging: The visions prospective cohort magnetic resonance imaging study*. Crit Care Med.

[R18] Murray C, Sanderson DJ, Barkus C, Deacon RM, Rawlins JN, Bannerman DM, Cunningham C (2012). Systemic inflammation induces acute working memory deficits in the primed brain: Relevance for delirium. Neurobiol Aging.

[R19] Nitchingham A, Caplan GA (2021). Current challenges in the recognition and management of delirium superimposed on dementia. Neuropsychiatr Dis Treat.

[R20] Oh ES, Fong TG, Hshieh TT, Inouye SK (2017). Delirium in older persons: Advances in diagnosis and treatment. Jama.

[R21] Reynish EL, Hapca SM, De Souza N, Cvoro V, Donnan PT, Guthrie B (2017). Epidemiology and outcomes of people with dementia, delirium, and unspecified cognitive impairment in the general hospital: Prospective cohort study of 10,014 admissions. BMC Med.

[R22] Shenkin SD, Fox C, Godfrey M, Siddiqi N, Goodacre S, Young J, Anand A, Gray A, Hanley J, MacRaild A (2019). Delirium detection in older acute medical inpatients: A multicentre prospective comparative diagnostic test accuracy study of the 4at and the confusion assessment method. BMC medicine.

[R23] Taylor J, Parker M, Casey CP, Tanabe S, Kunkel D, Rivera C, Zetterberg H, Blennow K, Pearce RA, Lennertz RC (2022). Postoperative delirium and changes in the blood-brain barrier, neuroinflammation, and cerebrospinal fluid lactate: A prospective cohort study. Br J Anaesth.

[R24] Thau L, Gandhi J, Sharma S (2023). Physiology, cortisol. Statpearls. Treasure Island (FL) ineligible companies. Disclosure: Jayashree Gandhi declares no relevant financial relationships with ineligible companies. Disclosure: Sandeep Sharma declares no relevant financial relationships with ineligible companies.

[R25] van Lieshout C, Schuit E, Hermes C, Kerrigan M, Frederix GWJ (2022). Hospitalisation costs and health related quality of life in delirious patients: A scoping review. Z Evid Fortbild Qual Gesundhwes.

[R26] van Montfort SJT, van Dellen E, Stam CJ, Ahmad AH, Mentink LJ, Kraan CW, Zalesky A, Slooter AJC (2019). Brain network disintegration as a final common pathway for delirium: A systematic review and qualitative meta-analysis. Neuroimage Clin.

[R27] Webber C, Watt CL, Bush SH, Lawlor PG, Talarico R, Tanuseputro P (2021). Hospitalization outcomes of delirium in patients admitted to acute care hospitals in their last year of life: A population-based retrospective cohort study. J Pain Symptom Manage.

[R28] Wilson JE, Mart MF, Cunningham C, Shehabi Y, Girard TD, MacLullich AMJ, Slooter AJC, Ely EW (2020). Delirium. Nat Rev Dis Primers.

[R29] Yu M, Li Y, Li B, Ge Q (2023). Inflammatory biomarkers and delirium: A mendelian randomization study. Frontiers in Aging Neuroscience.

